# Genome-wide identification, characterization and expression profile analysis of expansins gene family in sugarcane (*Saccharum* spp.)

**DOI:** 10.1371/journal.pone.0191081

**Published:** 2018-01-11

**Authors:** Thaís R. Santiago, Valquiria M. Pereira, Wagner R. de Souza, Andrei S. Steindorff, Bárbara A. D. B. Cunha, Marília Gaspar, Léia C. L. Fávaro, Eduardo F. Formighieri, Adilson K. Kobayashi, Hugo B. C. Molinari

**Affiliations:** 1 Embrapa Agroenergia. Parque Estação Biológica, Av. W3 Norte (final), Asa Norte, Brasília, DF, Brazil; 2 Instituto de Botânica, Núcleo de Pesquisa em Fisiologia e Bioquímica, São Paulo, SP, Brazil; Iowa State University, UNITED STATES

## Abstract

Expansins refer to a family of closely related non-enzymatic proteins found in the plant cell wall that are involved in the cell wall loosening. In addition, expansins appear to be involved in different physiological and environmental responses in plants such as leaf and stem initiation and growth, stomata opening and closing, reproduction, ripening and stress tolerance. Sugarcane (*Saccharum* spp.) is one of the main crops grown worldwide. Lignocellulosic biomass from sugarcane is one of the most promising raw materials for the ethanol industry. However, the efficient use of lignocellulosic biomass requires the optimization of several steps, including the access of some enzymes to the hemicellulosic matrix. The addition of expansins in an enzymatic cocktail or their genetic manipulation could drastically improve the saccharification process of feedstock biomass by weakening the hydrogen bonds between polysaccharides present in plant cell walls. In this study, the expansin gene family in sugarcane was identified and characterized by *in silico* analysis. Ninety two putative expansins in sugarcane (SacEXPs) were categorized in three subfamilies after phylogenetic analysis. The expression profile of some expansin genes in leaves of sugarcane in different developmental stages was also investigated. This study intended to provide suitable expansin targets for genetic manipulation of sugarcane aiming at biomass and yield improvement.

## Introduction

Plant cell walls are dynamic structures that determine and maintain the size and the shape of the cells and serve as a protective barrier. The cell walls are highly complex structures composed mainly of polysaccharides that vary in structure, function and abundance. In addition, cell walls are also composed of proteins, some of them with properties and functions not completely understood. Expansins are cell wall proteins discovered in cucumber hypocotyls that act in the remodeling of the plant wall by breaking the non-covalent links between cellulose microfibrils and polymers in the extracellular matrix in a pH dependent manner, leading to loosening, remodeling, assembly and cell extension [1, 2]. Furthermore, different studies have shown that expansins also influence plant biotic and abiotic stress relationship [2]. These cell wall proteins belong to a large superfamily that encodes proteins with size ranging from 225–300 amino acid residues, and are divided into four subfamilies: α-expansin (EXPA), β-expansin (EXPB), expansin-like A (EXPLA) and expansin-like B (EXPLB), based on phylogenetic relationship [3].

Plant expansins consist of two domains. The N-terminus domain (D1) is a six-stranded double-psi beta-barrel (DPBB) and it is characterized by the His-Phe-Asp motif and some conserved polar residues structurally similar to that of the family 45 of glycosyl hydrolases (GH45). The second domain (D2; so-called Pollen_allerg) contains conserved aromatic amino acids suitable for polysaccharide binding and aligned on the surface of a β-sandwich fold that resembles the motifs related to the family 63 of carbohydrate binding module domain (CBM63) [4]. The subfamily EXPA (α-expansis) are usually mediators of acid-induced cell wall loosening, while subfamily EXPB (β-expansins) comprise a subset of proteins known as group-1 grass pollen allergens as well as some other proteins not so well defined [5, 6]. In addition, expansins also comprise two smaller families, expansin-like A and B (EXPLA; EXPLB).

Some studies characterized expansins in plant genomes of angiosperms (Arabidopsis, poplar, grape, soybean, apple, Chinese cabbage, rice and maize) and nonflowering plants (*Selaginella moellendorffii* and *Physcomitrella patens*) (for a review, see [5]). Despite these efforts, the number of microbial expansins characterized and involved in disruption of cellulose fibers is greater compared with plants. Examples of plant expansins increasing the efficiency of cellulose hydrolysis are cucumber Ex29/Ex30, maize EXPB1 and tomato *Le*EXP2 [7, 8]. There had been few functional studies on plant expansins because of this multigenic family and the formation of inclusion bodies during heterologous expression in bacteria [9, 10].

The plant cell wall comprises one of the largest components of the earth´s biomass and many of its compounds are used in a variety of commercial products and industrial processes such as the textile industry, paper products, biofuels, food and more. In this context, it is thought that plant expansins could be manipulated to alter the structure of cell walls, increasing the added-value of the products derived from plant cell wall. Several studies have shown the action of expansins in leaf initiation and development [2]. For instance, Kuluev et al. [11] reported that overexpression of *Nt*EXPA5 increased the size of tobacco leaf and stems cells. Moreover, other recent studies have implicated expansins on leaf development initiation and growth in different plants such as Arabidopsis [12], wheat [13] and rice [14]. Therefore, overexpression of expansins can be associated with other breeding strategies, such as biomass improvement of crops with commercial importance. Sugarcane is an important crop able to accumulate high levels of sucrose in its culms and therefore it is largely used for sugar production, being responsible for approximately 80% of the sugar produced worldwide [15]. In addition, sugarcane biomass has the potential to be converted into biofuels such as second-generation ethanol and other added-value products. Sugarcane-based ethanol is an alternative fuel to fossil origin, being consider a renewable source of energy. The use of ethanol is considered an alternative to fossil fuels due to economic aspects and to low environmental impact, reducing ~85% of emissions of greenhouse gas (GHG) [16, 17, 18]. In order to produce ethanol from sugarcane, it is important that its biomass could be easily accessible to hydrolytic enzymes, a task that can be achieved diminishing biomass recalcitrance through conventional breeding or genetic manipulation. In addition, efforts have been made to find and characterize accessory proteins with non-hydrolytic activities on crystalline cellulose but with synergic activity with other cell wall hydrolases. Some studies suggest that the loosening of cellulose microfibrils by proteins such as expansins, which facilitate the access of the enzymes to the inner portion of the fibers, could increase the surface area available for the hydrolytic enzymes, establishing a more efficient process for biomass saccharification [2]. Currently, the sucrose accumulated in sugarcane culms is fermented into ethanol by yeasts, generating the so-called first generation ethanol (1G-bioethanol). The remaining biomass from this process composed of sugarcane bagasse, straw and senescing leaves is considered waste by the sugarcane industry, but may be used for the production of 2G-bioethanol [19]. Thus, identifying expansins responsible for sugarcane leaf growth could lead to a strategy to increase leaf biomass, therefore increasing the raw material to be converted into 2G-bioethanol.

The sugarcane genome was recently published [20], making genomic studies of this species more suitable. Here, we identified 92 expansins genes in the whole genome of sugarcane (cultivar SP80-3280). In addition, phylogenetic, structural and promoter sequence analyses were performed. Publically available transcriptome dataset and qPCR analyses were employed to study the expression of the identified genes at four different parts of leaves. The identification and expression analysis of expansins will assist in the use of these proteins in sugarcane to facilitate cellulose hydrolysis, increase plant biomass and produce bioethanol in a more efficient way.

## Materials and methods

### Identification of expansin genes in sugarcane

Sugarcane whole genome sequence (cultivar SP80-3280) was downloaded from GenBank with accession number GCA_002018215.1 to identify potential expansins. Posteriorly, 1392 amino acids sequences of plant expansins available in the National Center for Biotechnology Information (NCBI) bank were used as query to search for expansins in the genome sequence database using tblastn, with *e*-value of 10^−10^ as threshold, to identify sequences with high similarity. A custom script was developed and executed to remove redundant sequence and retrieve the similar sequence regions from sugarcane genome. The putative expansin sequences were analyzed to verify the presence of the signal peptide and Pollen allergen conserved domain (PF01357) through Pfam server (http://pfam.xfam.org/). The coding and complete nucleotide/amino acid dataset were used for further analysis. Molecular weight (MW), theoretical isoelectric point (pI) and protein length (aa) were characterized using the online tool Protparam (http://web.expasy.org/protparam/). Position of the signal peptide and PF01357 were predicted in the SignalP 4.1 (http://www.cbs.dtu.dk/services/SignalP/). Cellular function and gene ontology (GO) categories were determined using ProtFun2.2 Server (http://www.cbs.dtu.dk/services/ProtFun/).

To investigate the phylogenetic relationship and classification of sugarcane expansin (SacEXP) in α (EXPA), β (EXPB), like- A (EXPLA) and like-B (EXPLB), ten reference sequences (XP008373652, Q850K7, XP002278917, Q10S70, Q7XCL0, Q7XT40, Q9SHY6, Q0DZ85, AGM16366 and O80622) from all four expansin groups were used for phylogenetic analysis (**Table 1**). The reference sequences were collected from recent publications and used to classify the SacEXP. The proteins sequences were aligned with MUSCLE and the phylogenetic tree was built using FastTree 2.1.5 program [21]. The tree was visualized with the FigTree v.1.3.1 software.

**Table 1:** Number of expansin genes and their classification in sugarcane and other seven different species.

Species	EXPA	EXPB	EXPLA	EXPLB	Total
Sugarcane	51	38	3	0	92
Arabidopsis	26	6	3	1	36
Soybean	49	9	2	15	75
Rice	34	19	4	1	58
Maize	36	48	4	0	88
Tobacco	36	6	3	7	52
Tomato	25	8	1	4	38
Potato	22	6	3	8	39

### Structure analysis of SacEXP and promoter sequence

The sub-cellular localization of SacEXP was predicted with ProtComp 9.0 program (http://www.softberry.com/berry.phtml?topic=protcomppl&group=programs&subgroup=proloc) and the evolutionary conserved motifs were analyzed using Multiple Em for Motif Elicitation (MEME Suite 4.11.1) server software [22]. The number of motifs was set to eight. The dbCAN pipeline [23] was used to identify the possible protein classes involved in carbohydrate degradation from plant cell wall (CAZy - http://www.cazy.org/). The position and number of introns/exons of SacEXP were obtained by Gene Structure Display Server (GSDS 2.0).

### Promoter sequence analysis

A 1,000 bp upstream region from the initial codon of each SacEXP gene was trimmed from the respective contigs using custom scripts in Perl language. Subsequently, *cis*-elements of each SacEXP promoter region were determined by PlantCARE program (http://bioinformatics.psb.ugent.be/webtools/plantcare/html/).

### *In silico* expression analysis of SacEXP in sugarcane leaf

The available transcriptome data of the sugarcane cultivar SP80-3280 [20] was downloaded from NCBI´s Short Read Archive (SRA) database (http://www.ncbi.nlm.nih.gov/sra). For RNAseq SacEXP analysis, the data selected referred to results from +1 leaf segments of sugarcane, with the different segments classified as Base “zero” (B0), Base (B), Middle (M) and Tip (T), according to Matiello et al. [24]. The transcriptome was mapped using the program Bowtie 2.0. The software RSEM (RNA-Seq by Expectation Maximization) was used in *de novo* assembly to quantify the RNA-seq reads self-normalized using FPKM values (mean fragments per kilobase of transcript per million mapped reads) [25]. Both programs were implemented in Trinity assembling software [26]. Transcriptome datasets were mapped on SacEXP using blastn with *e*-value of 10^−10^ as threshold. The expression of transcripts was visualized by a hierarchical clustered heat map performed in the MeV software (http://www.tm4.org/mev.html).

### Plant material, RNA extraction and qRT-PCR analysis

The same genotype (SP80-3280) was used to study the expression profile of some of the expansins in sugarcane. The plants were planted in December of 2016 and grown in experimental fields of Embrapa Agroenergia, localized in Brasília-DF, Brazil. After eight months, the +1 leaf and the pointer of sugarcane were collected. The +1 leaf was harvested, divided in four different portions as described above (B0, B, M and T according to Matiello et al. [24]), with 3 biological replicates. The samples were frozen immediately in liquid nitrogen, ground into a fine powder and stored at -80°C.

The total RNA of three biological replicates for each segment was extracted using Trizol reagent (Invitrogen, USA), according to the manufacturer's instructions. The quality and concentration of RNA were evaluated by gel electrophoresis and NanoDrop, respectively (Thermo Fisher Scientific). Subsequently, one μg of RNA of each sample was treated with DNase I (Invitrogen, USA) and SuperScript® III kit (Invitrogen) was used for cDNA synthesis. The qRT-PCR was conducted in a 96-well optical plate using SYBR Green Master Mix (Applied Biosystems) and the reactions were performed in ABI 7500 Real-Time System (Applied Biosystems QuantStudio® 3) using 1 μL of diluted cDNA (1:25) and 0.2 μM of forward and reverse primers, in a final volume of 10 μL. Ten primers were designed using the software Primerquest (https://www.idtdna.com/primerquest/Home/Index). The expression level was normalized against the sugarcane GAPDH and EF1 genes by qRT-PCR. The thermal cycling used for amplifications was as follows: 2 min at 50°C min, 20 sec at 95°C, followed by 40 amplification cycles of 95°C for 3 sec, and 60°C for 30 sec using three biological and three technical replicates. The specificity of each reaction was verified through the dissociation curve profiles. Quantification cycle threshold (Cq) values per target were manually estimated. To calculate the relative expression level and primer efficiency estimation, background-corrected raw fluorescence data were imported into LinRegPCR version 2016.0 software [27]. The program uses linear regression analysis to fit a straight line and estimate PCR efficiency of each individual sample based on the slope of this line. The data were expressed as mean ± SEM. Statistical analysis of qPCR fold change in expression of genes among different segments were analyzed using one-way ANOVA test followed by the Turkey´s multiple comparation test. A p-value of 0.05 was considered to be significant.

## Results

### Identification and phylogenetic analysis of expansins in sugarcane

The genome of sugarcane cultivar SP80-3280 [20] was searched for putative expansin gene sequences based on high similarity with other previously identified expansins, detected in the web-based tools tblastn and Pfam.

The identified expansin genes were numbered sequentially and named with the prefix corresponding to the species, *Saccharum* spp. (SacEXPs). All putative expansins identified presented the signal peptide, the conserved double-psi β-barrel (DPBB) and the β-sandwich (D2 –Pollen allergen domain) domains, essential for its functioning [2]. We were able to find 89 sequences of candidate SacEXPs containing the His-Phe-Asp (HFD) motif, found only in α-expansin (EXPA) and β-expansin (EXPB) [28]. From these sequences, 51 contained large insertions (α-insertion) and deletions (β-insertion), characteristic of EXPA [29]. Furthermore, three additional sequences were found and characterized as expansin-like A (EXPLA), due to the presence of extension at the C-terminus and absence of the HDF domain [28]. These results suggest a total of 92 expansins or expansin-like sequences in sugarcane, from which 51 were EXPA, 38 EXPB and 3 EXPLA **(S1 Table** and **S1 Fig)**. EXPLB could not be found in the sugarcane genome (**S1 Fig**). The number of EXPA, EXPB, EXPLA and EXPLB found in sugarcane was compared with other available characterized expansins from dicot (Arabidopsis, soybean, tobacco, tomato and potato) and monocot (rice and maize) plants. As observed, the sugarcane genome contains a larger number of expansins compared to other species such as Arabidopsis (36), soybean (75), rice (58), maize (88), tobacco (52), tomato (38) and potato (39) (**Table 1)**. All references related to each of these studies can be found in a recent review by Cosgrove [5]. The sequences identified in the present study were deposited in the GenBank database (access MG204112-MG204203) (**S1 Table**).

**Fig 1:**
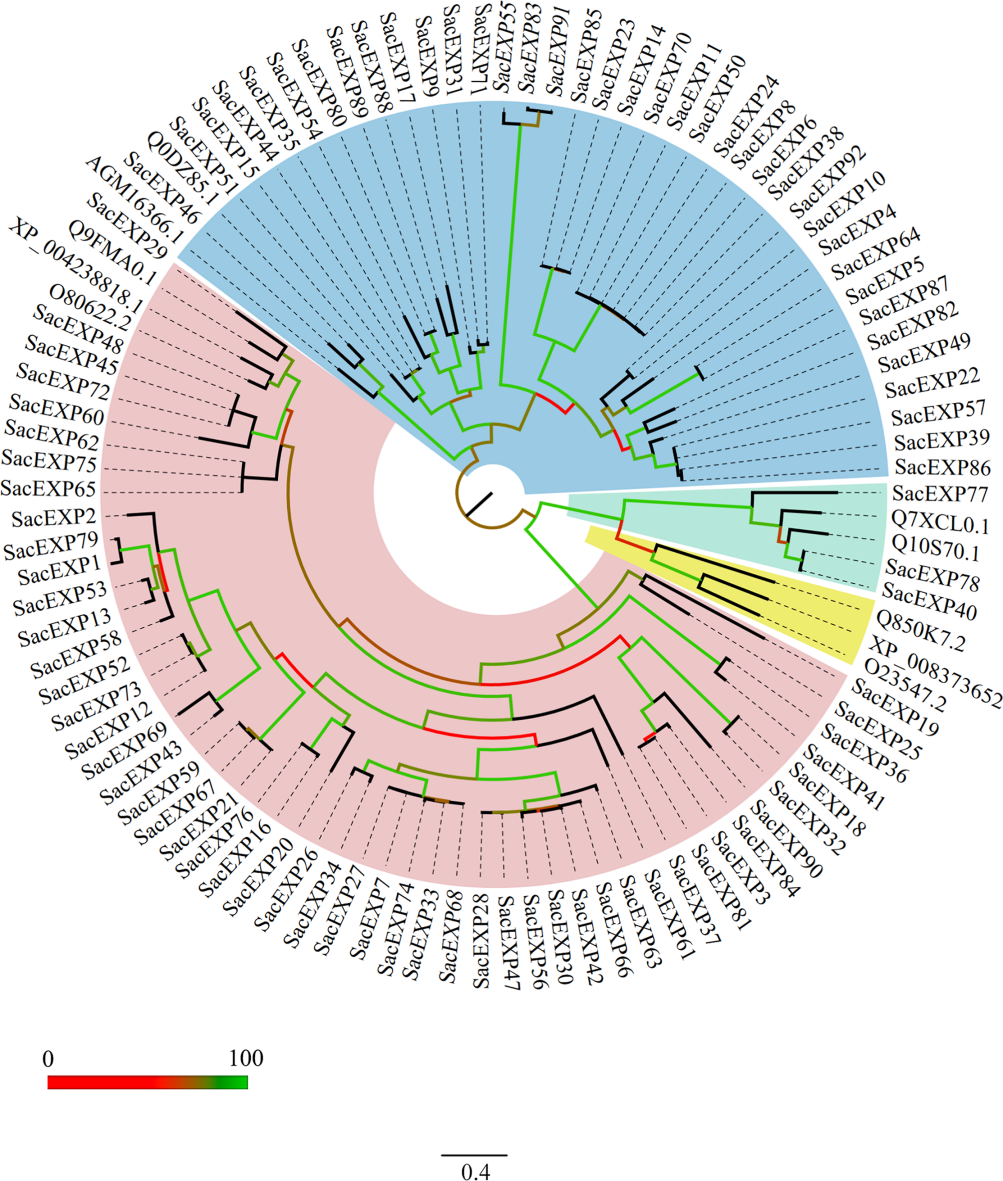
Maximum likelihood phylogeny of 92 sugarcane SacEXP amino acid sequences. The classification of expansins is represented by different colors: Pink—EXPA; Blue—EXPB; Green—EXPLA; Yellow—EXPLB. Branch color scale represents SH-like local support (red to lower values and green to higher values).

The sequences of the sugarcane expansin family and the reference sequences were aligned and a phylogenetic tree was generated (**Fig 1**). According to the evolutionary relationship of the unrooted tree, the 92 expansins were divided into three subfamilies: 51 α-expansins (EXPA), 38 β-expansins (EXPB) and 3 expansin-like A (EXPLA), with SH-like local support values based on branch color scale (http://www.microbesonline.org/fasttree). As mentioned above, expansin-like B (EXPLB) was not detected in sugarcane. The phylogenetic data generated for sugarcane confirm the structural differentiation of expansin gene superfamily. A second phylogenetic tree containing sequences of maize, Arabidopsis and rice was generated to track the evolutionary history of SacEXPs (**S2 Fig**). As expected, the sequences of SacEXP were more closely related to the maize (*Zm*EXP) and rice (*Os*EXP) than to Arabidopsis (*At*EXP) expansins.

### Structural analysis of SacEXP genes

**Fig 2:**
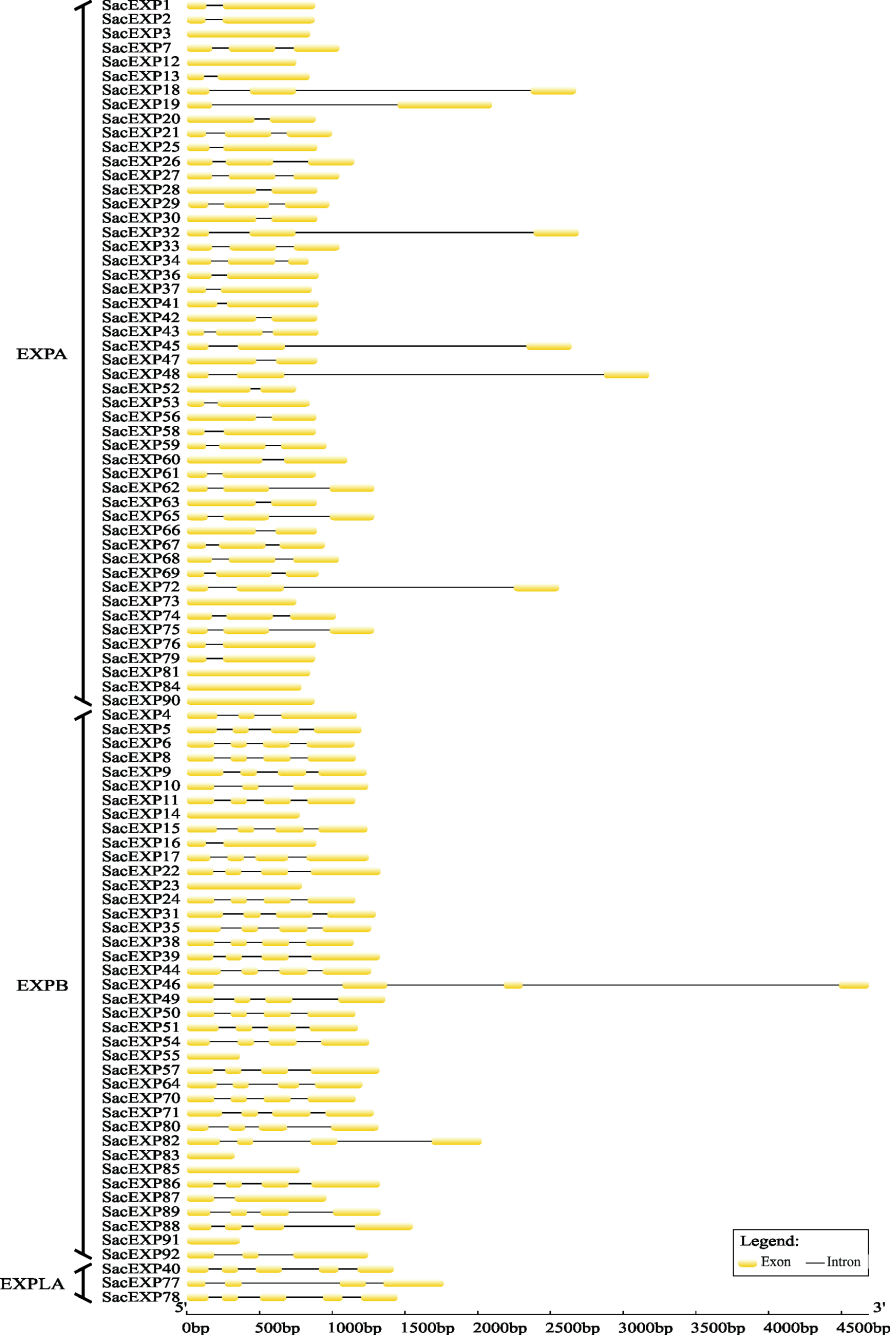
Intron/exon pattern of SacEXP gene. Exons and introns are shown as yellow boxes and thin lines, respectively.

**S1 Table** shows some properties of sugarcane expansins. The SacEXPs size ranged from 109 to 317 aa, with MW ranging from 11.31 to 34.57 KDa and pI from 4.88 to 9.75. The signal peptide length is comprised of 15 to 29 aa and the Pollen allergen (D2) domain about 34–87 aa. In general, expansins encode small proteins ranging from 225 to 300 aa [2]. Apparently, in the sugarcane genome, expansin deletions occurred in SacEXP34, SacEXP55, SacEXP83 and SacEXP91, because these proteins vary from 109 to 209 aa, while insertions appeared to occur in SacEXP17, SacEXP22, SacEXP31, SacEXP39, SacEXP57, SacEXP60, SacEXP71 and SacEXP86, since these expansins have between 307 and 317 aa. Comparative structural analysis of exons-introns revealed that, in general, SacEXP genes have 1 to 3 introns (**Fig 2**). Each group has a similar organization pattern, with the predominance of 1 to 2 introns in EXPA and 3 introns in EXPB and EXPLA, but SacEXP40 (EXPLA) presented 4 introns. Genes containing only one intron encode for small proteins compared to the others (**Fig 2**).

To investigate the subcellular localization of expansins, we used the ProtComp to search for localization-specific motifs. The analysis suggested that all sugarcane expansins identified appear to be secreted extracellularly. In addition, analysis of the CAZy distribution for sugarcane expansin superfamily showed that 19 expansins (SacExp4, SacExp7, SacExp15, SacExp22, SacExp26, SacExp27, SacExp33, SacExp35, SacExp36, SacExp39, SacExp41, SacExp44, SacExp54, SacExp57, SacExp68, SacExp74, SacExp80, SacExp86 and SacExp89) were classified with the carbohydrate binding module CBM63, which exhibits a high specificity to cellulose binding (**Table 2**). Out of these 19 proteins, eight were classified as EXPA and 11 as EXPB. All other sugarcane expansins do not appear to have the carbohydrate binding domain CBM63 (**Table 2**).

**Table 2:** Determination of carbohydrate binding module (CBM) position using dbCAN HMM 5.0.

Expansins	Module	Subfamily	Position
SacEXP4	CBM63	EXPB	182–256
SacEXP7	CBM63	EXPA	183–245
SacEXP15	CBM63	EXPB	183–259
SacEXP22	CBM63	EXPB	171–244
SacEXP26	CBM63	EXPA	199–249
SacEXP27	CBM63	EXPA	183–245
SacEXP33	CBM63	EXPA	183–245
SacEXP35	CBM63	EXPB	192–268
SacEXP36	CBM63	EXPA	176–243
SacEXP39	CBM63	EXPB	171–244
SacEXP41	CBM63	EXPA	189–256
SacEXP44	CBM63	EXPB	192–268
SacEXP54	CBM63	EXPB	166–240
SacEXP57	CBM63	EXPB	171–244
SacEXP68	CBM63	EXPA	183–245
SacEXP74	CBM63	EXPA	183–245
SacEXP80	CBM63	EXPB	188–238
SacEXP86	CBM63	EXPB	171–244
SacEXP89	CBM63	EXPB	169–242

As described above, expansins usually contain two conserved domains (DPPB-1 and Pollen_Allerg-D2), but these proteins can also present other functional motifs. Using the web-based tool MEME [22], seven conserved motifs in SacEXP were identified (**S3 Fig**). All 92 expansins contain three conserved motifs (motifs 1, 2 and 4 in the **S3 Fig**), although the type and location may change. Analysis of Pfam revealed that the signal peptide is included in motif 1, the DPPB_1 domain was found in motif 2 or 3 and the Pollen_allerg (D2) domain was localized in motif 4 or 5 (**S3 Fig**).

### GO analysis

**Fig 3:**
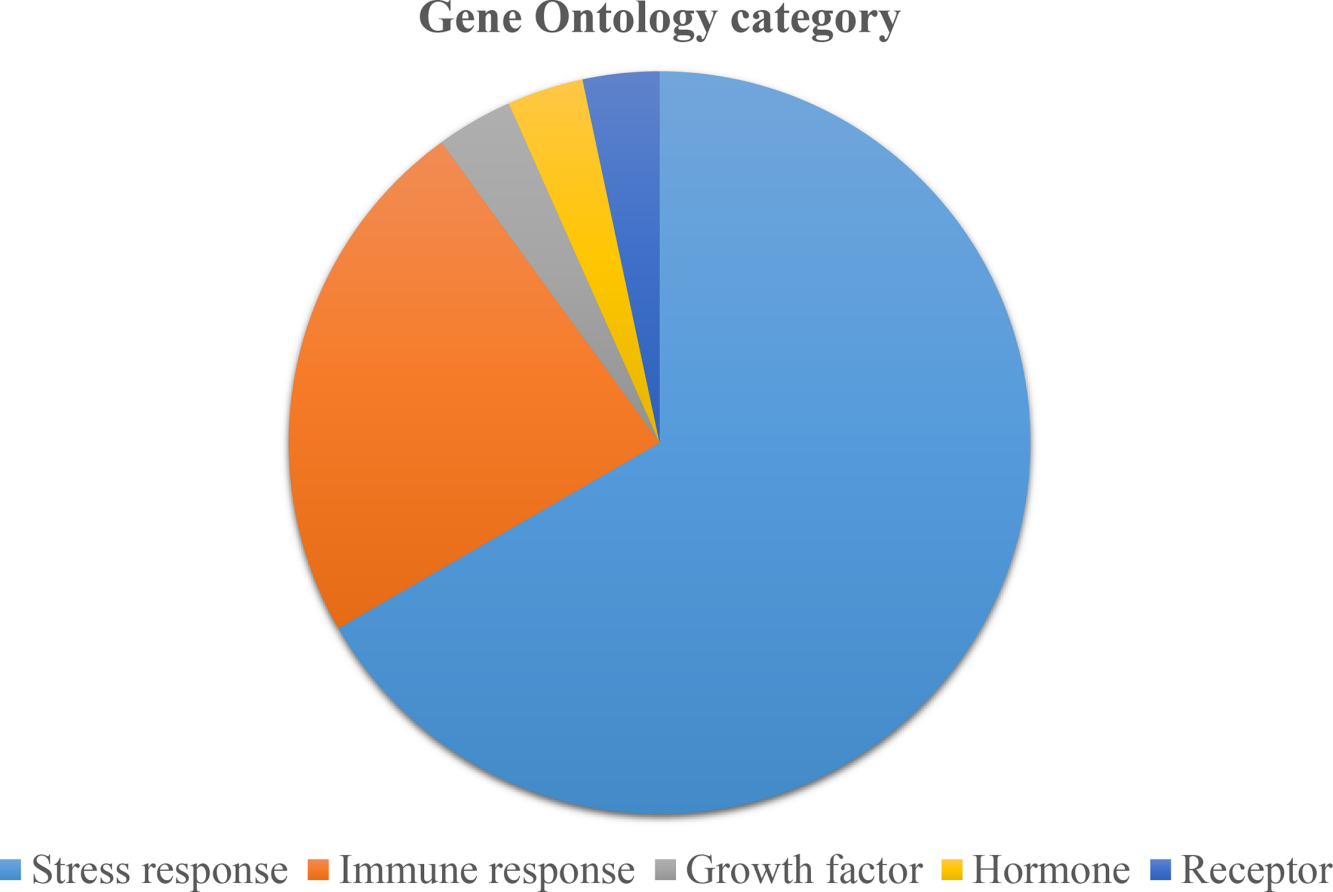
Gene ontology category of SacEXP determined using ProtFun.

To predict the cellular and/or biological roles of SacEXP, GO category annotation was analyzed by employing the ProtFun server. In SacEXPs, with exception of SacEXP29, included in transport and binding category, all others SacEXPs were classified into the cell envelope functional category. The most enriched GO categories were stress response (67% of SacEXPs). Immune responses (23%) and receptor, hormone and growth factor categories (3.3%) (**Fig 3**).

### *Cis*-elements in SacEXP gene promoters

The presence of putative *cis*-elements in sugarcane expansin genes was also investigated. The *cis*-elements were divided into classes responsive to stress, hormone and light as described by Feng et al. [30]. Some of the stress responsive *cis-*elements in SacEXP promoters were related with heat, drought, low-temperature, defense mechanism, anaerobic condition and fungal elicitor. In addition, *cis*-elements related to salicylic acid (SA), methyl jasmonate (MeJA), gibberellins (GA), auxin (IAA) and ethylene were also identified. The *cis*-elements more frequently found in sugarcane expansin promoters were associated with light regulation and MeJA (**S2 Table**).

### Expression analysis of SacEXP along the sugarcane leaf

**Fig 4:**
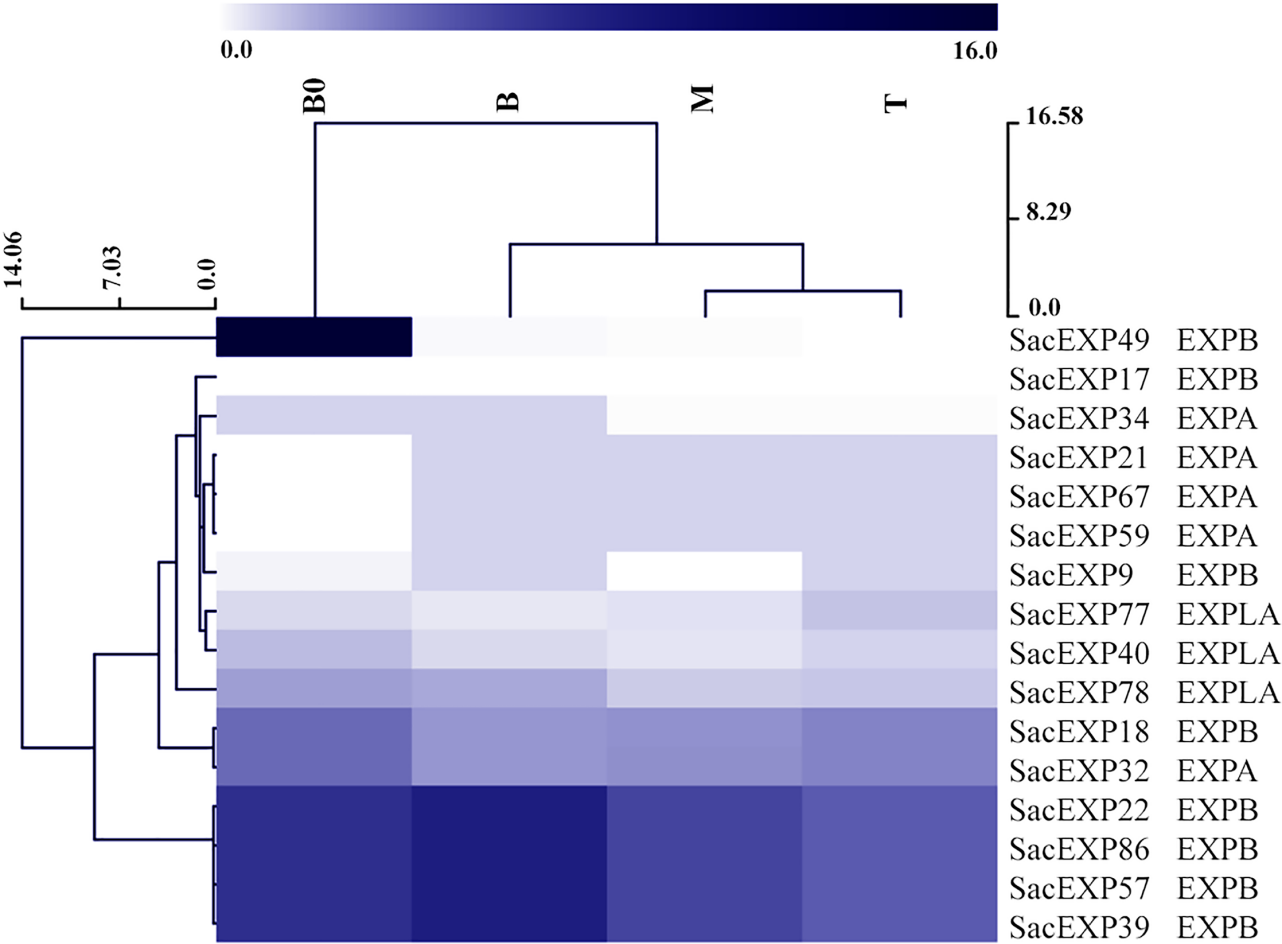
Expression pattern of SacEXP in different leaf segments: Base “zero” (B0), base (B), middle (M) and Tip (T) determined by *in silico* analysis.

Expansins are commonly involved in leaf initiation and growth [2], therefore, expansin gene expression was analyzed throughout the whole leaf of sugarcane. The leaves were divided in 4 different portions, classified as Base zero (B0), Base (B), Middle (M) and Tip (T). Subsequently, the expression pattern of expansins in leaves of 60 days-old sugarcane plants (cultivar SP80-3280) were analyzed using publically available transcriptomic data [24]. Out of the 92 expansins identified in the present study, the expression of sixteen expansin genes was observed in different portions of the leaves. The expansin gene expression profile in sugarcane leaf comprised 7 EXPB (SacEXPs 9, 17, 22, 39, 49, 57, and 86), 6 EXPA (SacEXPs 18, 21, 32, 34, 59 and 67) and 3 EXPLA (SacEXPLAs 40, 77 and 78), with the β-expansin genes 22, 39, 57 and 86 identified as the most expressed along the whole leaf **(Fig 4)**. These highly expressed expansin genes presented the CBM63 module, while in the other identified genes such module was absent (**Fig 4** or **Table 2**). In general, expansins demonstrated high expression levels at the B0 portion of the leaf, known as the region presenting a high number of meristematic cells and as a point of junction between the leaf and the stem, followed by the base portion (B), responsible for the expansion of a growing leaf. Interestingly, the α-expansins 21, 59 and 67 were not expressed at the B0 portion, but demonstrated constant levels of expression along the leaf portions. In addition, the SacEXP49 (β-expansin) presented the strongest and highest expression level at the B0 portion, with low or absent expression levels in the other leaf portions, making this expansin as an outlier in the *in silico* analysis. The expansin-like SacEXPLAs 40, 70 and 78 demonstrated intermediary levels of expression throughout the whole leaf **(Fig 4)**.

**Fig 5:**
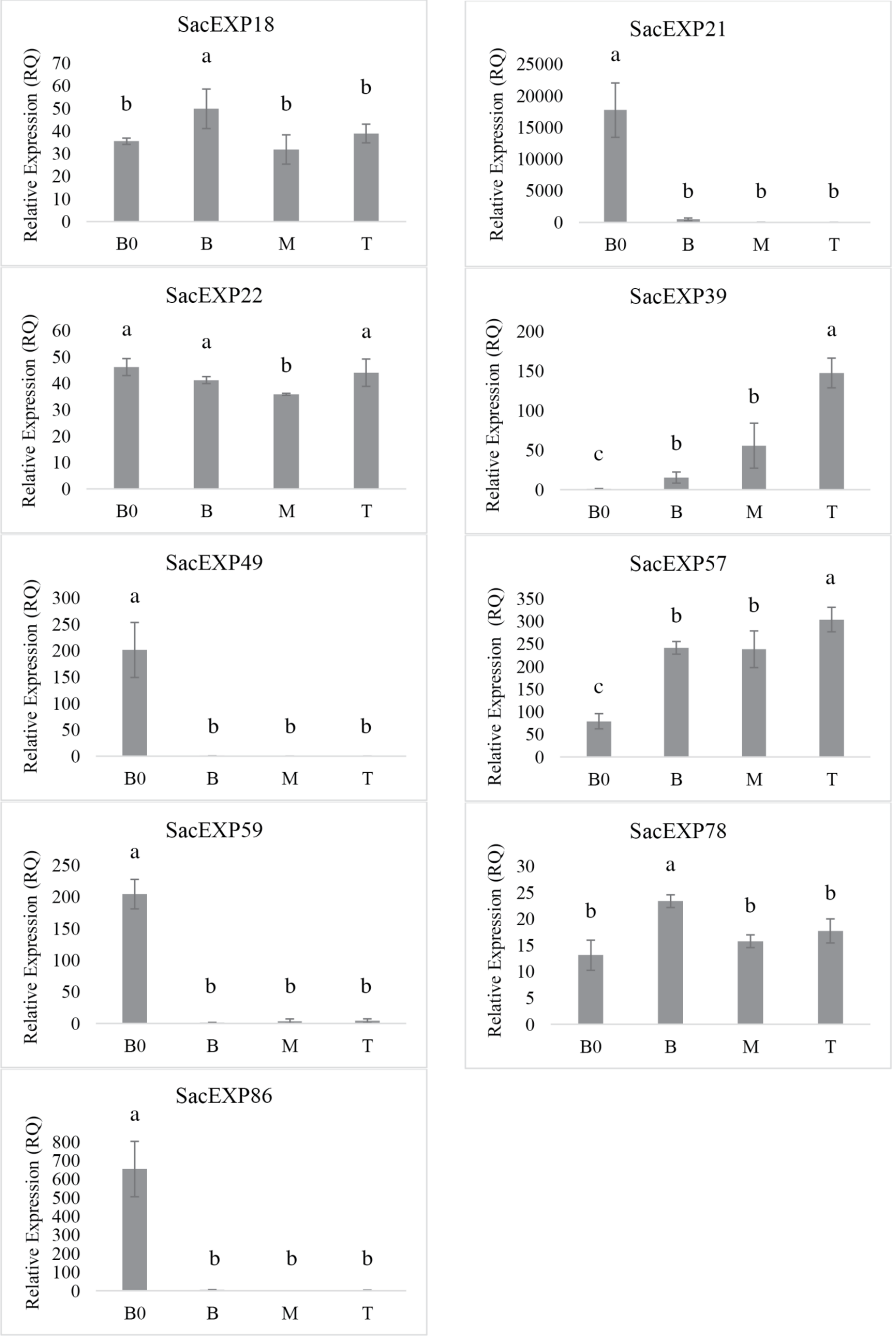
Expression profile of genes encoding expansins in segments of sugarcane leaf: Base "zero" (B0), Base (B), medium (M) and tip (T) determined by Mattielo et al. 2015. Values represent mean± standard error of the mean (SEM, n = 3) for each segments of sugarcane leaf. Individual bars with different alphabets indicate statistical significance (p<0.05).

**Fig 6:**
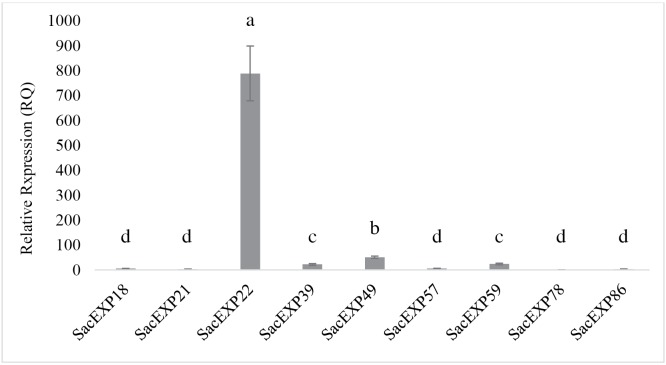
Expression profile of expansin genes in sugarcane index segment (culm pointer).

To verify whether the expression pattern of expansins would change during sugarcane development, qRT-PCR analyses were performed using leaf portions from 8-month-old plants (**S3 Table**). Out of the 16 expansins verified by *in silico* analysis, transcripts of nine expansins with high expression levels were detected in at least one portion of the leaf. As observed in **Fig 5**, the most expressed expansin revealed by qRT-PCR was the α-expansin SacEXP21, presenting high levels of expression only at the B0 portion of the leaf. SacEXP49 (EXPB) gene was also only expressed at the B0, but the levels of expression were lower compared to SacEXP21 gene. Corroborating the data obtained from *in silico* analysis, it was verified that the majority of the expansins studied presented high levels of expression at the B0 leaf portion, even at an older stage of development, with exception of SacEXP39 (EXPB), which was not expressed at this leaf portion. The genes SacEXP18 (EXPA) and SacEXP78 (EXPLA) were expressed at approximately the same levels along the leaf, and the genes SacEXP49, SacEXP59 and SacEXP86 were expressed exclusively at B0. To verify if these genes were expressed in sugarcane culms, the expression pattern of expansins in the culm tip (**Fig 6**) of 8-month-old sugarcane plants was analyzed. As shown in the **Fig 6**, the majority of SacEXPs expressed in leaves were also expressed in the culms. In general, β-expansin gene (SacEXP22, SacEXP49, SacEXP39) were the most SacEXPs expressed in sugarcane culms, demonstrate possibly tissue specialization. In summary, these results indicate that high levels of expansin expression are found in the meristematic region (B0) of a growing leaf and in the same region of mature leaves in sugarcane.

## Discussion

Expansins are a diverse family of proteins responsible for modification of plant cell walls and potentially involved in plant development and growth [2]. Despite being discovered two decades ago [1], the mechanisms through which expansins act to loosen the cell wall are still poorly understood. The expression and characterization of expansins have been reported in different species such as Arabidopsis, soybean, wheat, tobacco, tomato, grapevine and potato. In grasses, expansins were characterized in rice [31] and maize [32], but detailed studies in monocots are still needed. A distinctive feature of grasses is the presence of (glucurono)arabinoxylan in the cell walls and the cross-link of ferulate and *p*-coumarate between cellulose, hemicellulose and lignin polymers [33]. The unique composition of grass cell walls requires different mechanisms for cell wall remodeling, including the expansins repertoire, when compared to eudicot or other monocot plants. To gain more insight into grasses expansins, we performed *in silico* analysis to identify and characterize the expansin family of the important economic crop sugarcane (*Saccharum* spp.), which is among the most important crops worldwide. Sugarcane accumulates high content of sucrose in its culms and, therefore, it is used for sugar and ethanol (1G) production, being considered as an “energy” crop. The remaining biomass from the culm processing is mainly composed of bagasse, straw and leaves, which can be used as feedstocks for 2G-bioethanol and other added-value products [34]. Therefore, the identification and expression analysis of putative expansins could assist in the use of these proteins in sugarcane to facilitate the cellulose hydrolysis, increase plant biomass and produce bioethanol in a more efficient way.

Recently, the whole genome assembly of a highly productive commercial cultivar of sugarcane from Brazil (SP80-3280) became available, allowing the identification and characterization of new genes in this crop [20]. In this work, we were able to identify 92 putative expansins or expansin-like sequences in sugarcane (SacEXPs), from which 51 were classified as α-expansins (EXPA), 38 as β-expansins (EXPB) and 3 as expansin-like A (EXPLA). All putative identified SacEXPs presented the signal peptide, the conserved double-psi β-barrel (DPBB-D1) and the Pollen_allergen (D2) domains, characteristic of other expansins identified to date [2, 5, 35]. In addition, as observed by GO category annotation analysis, the majority of SacEXPs fell into the cell envelope functional category, as expected for their function on cell walls **(Fig 3)**. Previous studies with expansins have demonstrated that gene duplication is possibly responsible for the expansion of these gene subfamilies [36]. In our SacEXP analysis, seven genes are apparently associated with gene duplication, especially because of the combination of these genes within the same group in the phylogenetic analysis and the high similarity between them (SacEXP65/75, SacEXP59/67, SacEXP45/48, SacEXP30/42, SacEXP7/27/33, SacEXP6/8/11/24/50/70 and SacEXP3/81, **Fig 1** and **S1 Table**). Moreover, the diversity of EXPB sequences in sugarcane is consistent with the large number presented by other grasses such as rice, for which 18 EXPB were identified, in comparison with the eudicot Arabidopsis, which possess only 6 EXPB genes [37]. It is not well established when expansins appeared during the evolution of plants, but it is believed that EXPA and EXPB exist before the divergence between vascular plants and mosses and EXPLA and EXPLB just appeared in the ancestor of angiosperms and gymnosperms [37]. As pointed out by Sampedro et al. [6], the divergent EXPB proteins have evolved possibly to act on highly substituted xylans such as (glucurono)arabinoxylans, allowing more efficient roles in the cell wall mechanics of primary cells of grasses.

Out of the 92 proteins identified in sugarcane, CAZy distribution analysis demonstrated that 19 expansins presented the family 63 carbohydrate-binding module (CBM63; **Table 2**), which resembles a CBM of bacterial cellulases [4]. The CBM consists of catalytic domains in cellulolytic enzymes that potentiate enzyme activity by approximating and targeting cellulose [38]. Although the expansins have the domain resembling CBMs, it is usually thought that in these proteins the CBM is not loosely linked to a catalytic domain by a flexible linker as for classical CBMs. Probably, the CBM is tightly packed against another domain, favoring a coordinated movement able to promote a distortion in the cellulose chains, subsequently generating a mobile conformational defect that promotes cell wall loosening [35]. We were not able to find the CBM63 in all expansins identified in sugarcane. However, considering the distinctive nature of grasses cell walls, the presence of other polysaccharide-binding modules in SacEXPs should not be dismissed.

We also searched for putative *cis*-elements in SacEXP promoters and we identified a number of stress responsive, hormonal and light regulation *cis*-elements. In accordance with expansin promoters found for other species [39], the majority of SacEXP promoters appeared to have *cis*-elements associated with light regulation and hormones, especially MeJA **(S2 Table)**. It is not surprising that expansin gene promoters are enriched in *cis*-elements responsive to light since it is known that leaf and shoot expansion changes in a time-of-day-dependent manner [40]. Moreover, expansin genes expression have been shown to control cell wall extensibility during shade avoidance in *Stellaria longipes* [41]. It is also expected for expansin promoters to have *cis*-elements responsive to hormones as many studies demonstrated that the action of EXPs can be influenced by hormones such as cytokinins [42], ethylene [43] and auxins [1, 44]. The enrichment of promoters containing MeJA-responsive *cis*-elements in SacEXP genes might not be ruled out at this point and it is an interesting question to be addressed in future research, once it is known that MeJA is involved in plant growth and development [45].

As described above, sugarcane leaves are attractive targets for genetic manipulation mainly because these tissues can be used as biomass to be converted in 2G-bioethanol. Therefore, increasing sugarcane leaf biomass could be a strategy to improve the generation of biofuels. Leaf and stem (culm) expansion is pivotal for yield in sugarcane [46], and it is not surprising that expansins could be suitable targets for genetic manipulation in sugarcane to improve yield. That said, we analyzed SacEXPs expression profile along the sugarcane leaf blade at two different stages of sugarcane development. First, using publicly available data [24], we could find the expression of 16 expansins throughout the leaves of young, fast-growing 2-months old plants (five EXPA, eight EXPB and three EXPLA; **Fig 4**). Based on our analysis, we could not find a regular pattern of SacEXP expression along the leaf. In general, higher levels of expression were observed at the B0 portion of the leaf, which is the portion presenting a high number of meristematic cells and as a point of junction between the leaf and the stem, followed by the base portion, responsible for the expansion of a growing leaf. These results are somehow expected since these leaf portions mostly contain active expanding cells [46]. An interesting result was related to the expression of the α-SacEXPs 21, 59 and 67, which were not expressed at the B0 portion, but was constantly expressed along the other leaf portions. The differential expression of SacEXP genes in the sugarcane leaf suggests that the composition of the cell wall might be different along the leaf portions, therefore requiring a different repertoire of expansins in order to expand or respond to environmental signals.

To verify if the expression pattern of expansins would change during sugarcane development, it was performed qRT-PCR using leaves from 8-month-old plants, a developmental stage associated to pre-harvesting (ripening) in sugarcane. From the 16 expansins expressed in younger leaves, only nine SacEXP were expressed in leaves of ripening sugarcane **(Fig 5)**. The expression patterns of these genes were slightly different in mature sugarcane when compared with younger plants. Only SacEXP18, SacEXP49 and SacEXP78 presented the same expression pattern in both developmental stages. In leaves of mature sugarcane, SacEXP21 (EXPA) was the most expressed expansin at the B0 portion, but in the same portion of 2-month-old plants, the most expressed gene was SacEXP49 (EXPB). At this point, we cannot rule out the reasons for these discrepancies, since more biochemical and functional genomics studies are needed to address these issues. Despite of the irregular expression pattern in leaves of sugarcane from different developmental stages, our results suggest that in general SacEXPs appeared to be highly expressed in active, fast growing cells at the B0 leaf portion. However, it should be taken into consideration that expansin gene expression is a highly regulated process responding to different physiological and environmental stimulus.

This study intended to provide suitable expansin targets for genetic manipulation of sugarcane aiming at biomass and yield improvement. In addition, genetic manipulation of expansins might lead to resistance to abiotic and biotic stresses since the expansins are known to be involved in different physiological processes such as stomata opening and closing, reproduction, ripening and stress tolerance (for review see Marowa et al. [2]). The data presented here demonstrate that expansins are differentially regulated even in different portions of the same tissue, indicating that genetic manipulations should be handled with caution in order to avoid pleiotropic effects and consequent yield losses.

## Supporting information

S1 Fig α- insertion and β-insertion localized within SacEXPs. (TIF)

S2 Fig Evolutionary phylogenetic tree of wheat (TA), *Arabidopsis* (AT) and sugarcane (Sac). Green, expansin-like A (EXPLA); red, α-expansins (EXPA); blue, β-expansins (EXPB).
(TIF)

S3 Fig Eight conserved motifs of SacEXP proteins according to MEME software. Different color boxes correspond to different motifs. For details, see Material and methods.
(TIF)

S1 Table Characterization of SacEXP gene from *Saccharum* spp. aa, lenght of aminoacids; N° Intron; MW, molecular weight; pI, isoelectric points; PSI, position signal peptideo; PPA, position pollen allerg.
(DOCX)

S2 Table *Cis*-elements in the promoter of SacEXP genes related with anaerobic induction, fungal elicitor, drought, defense and stress, heat, low temperature, salicilic acid (SA), auxin (IAA), gibberlin (GA), methyl jasmonate (MeJA), ethylene, abscisic acid (ABA) and light. (DOCX)

S3 Table Gene-specific SacEXP primers. (DOCX)
