# Correction: Genome-wide identification, characterization and expression profile analysis of expansins gene family in sugarcane (*Saccharum* spp.)

**DOI:** 10.1371/journal.pone.0196140

**Published:** 2018-04-17

**Authors:** Thaís R. Santiago, Valquiria M. Pereira, Wagner R. de Souza, Andrei S. Steindorff, Bárbara A. D. B. Cunha, Marília Gaspar, Léia C. L. Fávaro, Eduardo F. Formighieri, Adilson K. Kobayashi, Hugo B. C. Molinari

The following information is missing from the Funding section: LCLF also acknowledges BNDES FUNTEC Project Enzimas e leveduras para produção de etanol a-partir da biomassa da cana).
