# Supplementary figures and images for: Genome-wide identification, characterization and expression profile analysis of expansins gene family in sugarcane (Saccharum spp.)
Source: PLoS One. 2018 Jan 11;13(1):e0191081. doi: 10.1371/journal.pone.0191081 (PMC5764346; doi:10.1371/journal.pone.0191081)

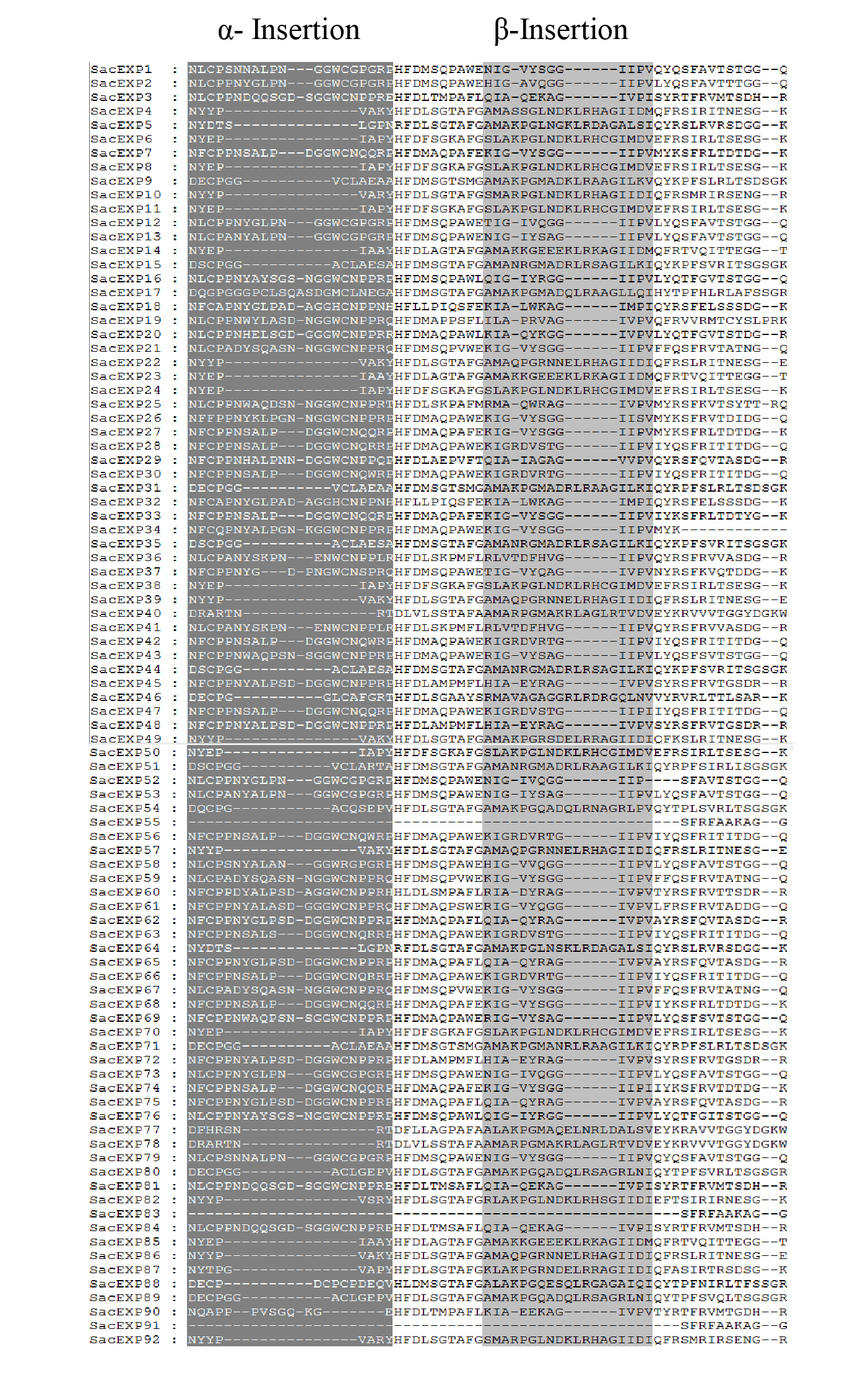

Supplement: S1 Fig — (TIF) [file pone.0191081.s001.tif]

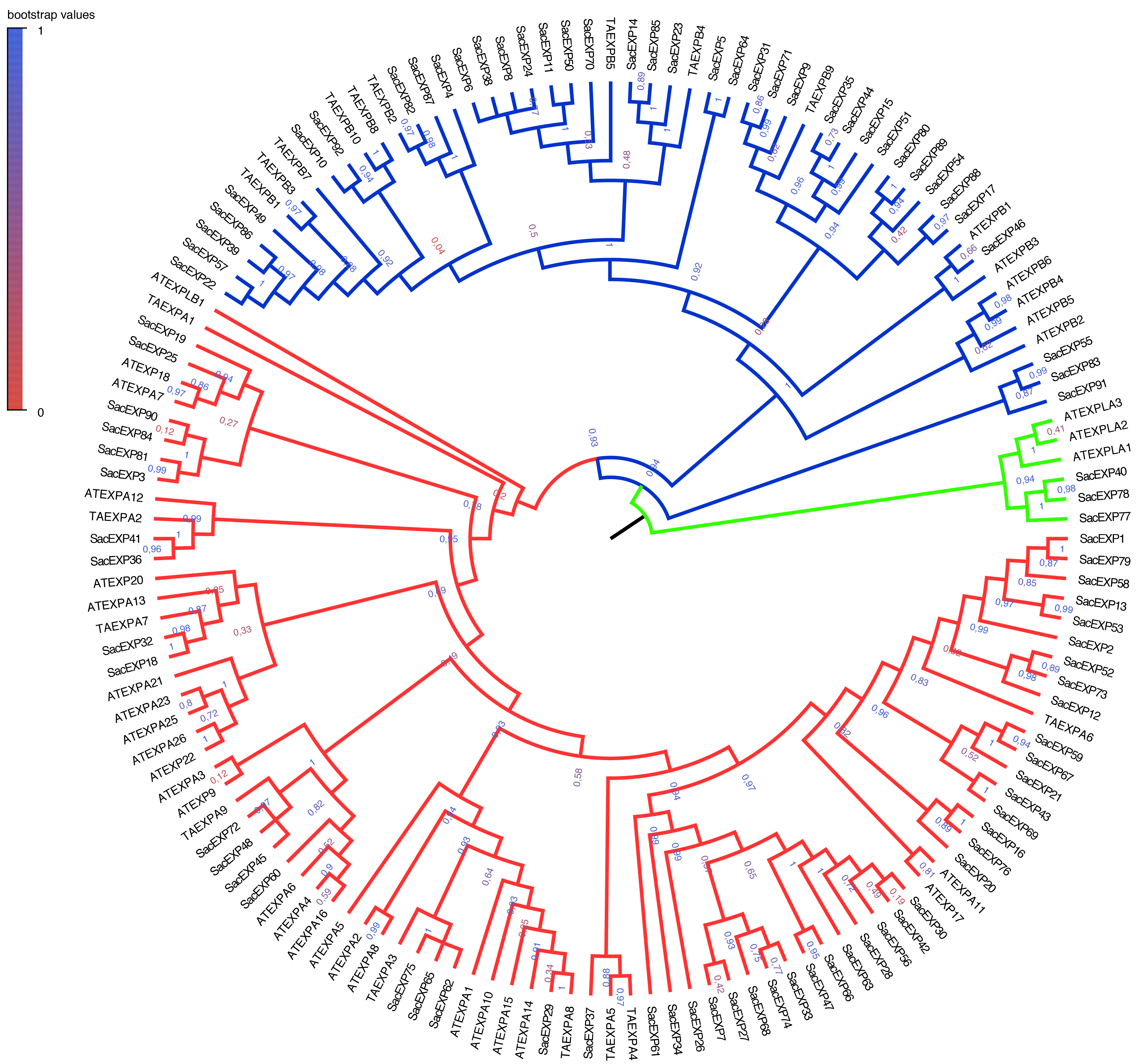

Supplement: S2 Fig — Green, expansin-like A (EXPLA); red, α-expansins (EXPA); blue, β-expansins (EXPB). (TIF) [file pone.0191081.s002.tif]

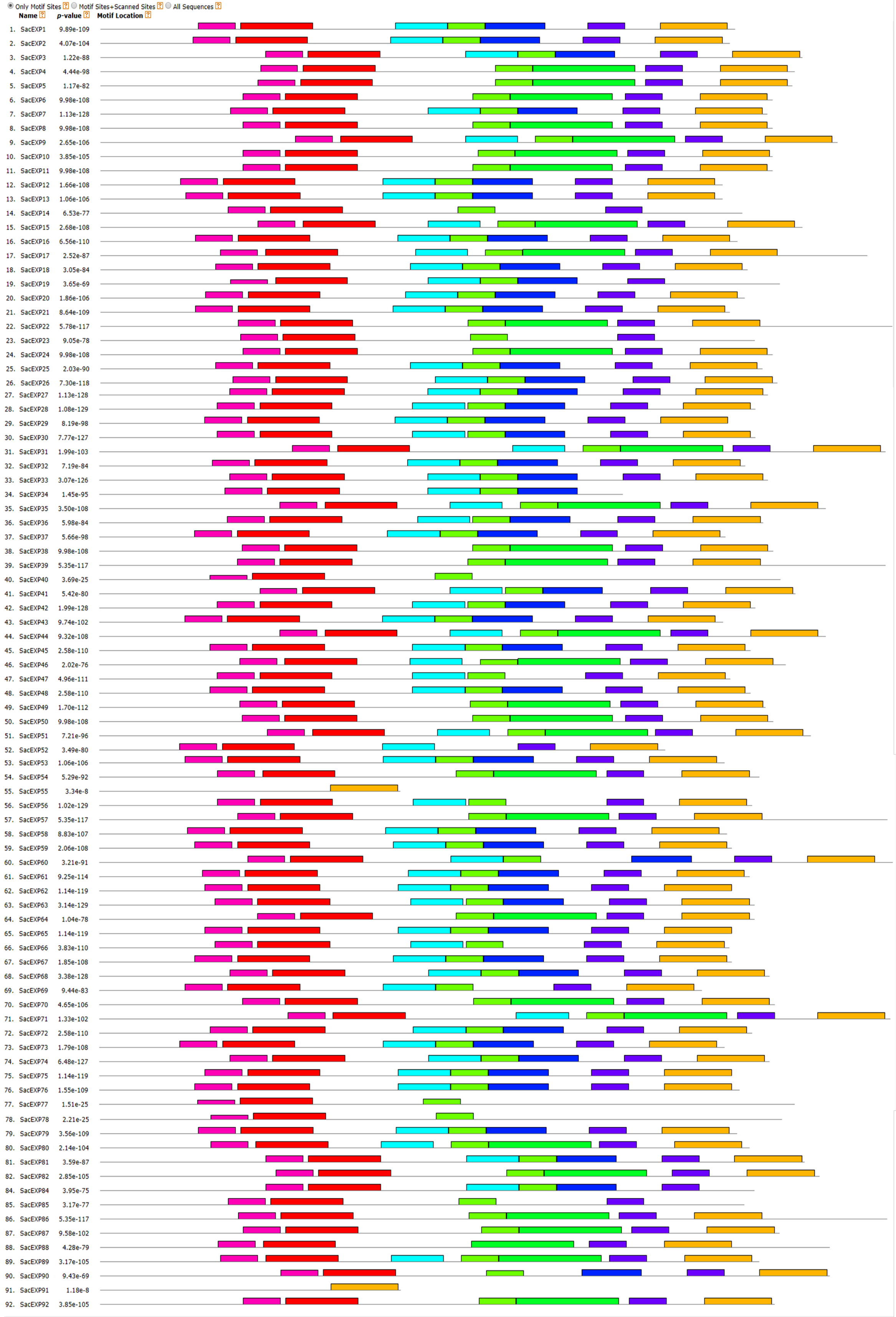

Supplement: S3 Fig — Different color boxes correspond to different motifs. For details, see Material and methods. (TIF) [file pone.0191081.s003.tif]
