# Supplementary material for: Genome-wide identification, characterization and expression profile analysis of expansins gene family in sugarcane (Saccharum spp.)
Source: PLoS One. 2018 Jan 11;13(1):e0191081. doi: 10.1371/journal.pone.0191081 (PMC5764346; doi:10.1371/journal.pone.0191081)
Supplement: S1 Table — aa, lenght of aminoacids; N° Intron; MW, molecular weight; pI, isoelectric points; PSI, position signal peptideo; PPA, position pollen allerg. (DOCX) [file pone.0191081.s004.docx]

| Expansin ID | aa |  | MW (kDA) | pI | PSP | PPA | Contig localization | GenBank ID |
| --- | --- | --- | --- | --- | --- | --- | --- | --- |
| SacEXP1 | 254 |  | 26.97 | 8.05 | 1-25 | 160-237 | SCSP803280_000000780,18701,19580 | MG204139 |
| SacEXP2 | 252 |  | 26.43 | 8.53 | 1-21 | 158-235 | SCSP803280_000000780,28358,29234 | MG204140 |
| SacEXP3 | 281 |  | 29.58 | 8.83 | 1-28 | 188-266 | SCSP803280_000006787,6156,7001 | MG204197 |
| SacEXP4 | 278 |  | 30.48 | 6.37 | 1-21 | 180-261 | SCSP803280_000064610,960,2126 | MG204192 |
| SacEXP5 | 277 |  | 29.88 | 9.48 | 1-24 | 180-261 | SCSP803280_000064610,15582,16780 | MG204193 |
| SacEXP6 | 269 |  | 29.16 | 8.96 | 1-25 | 171-253 | SCSP803280_000008918,18667,19817 | MG204165 |
| SacEXP7 | 267 |  | 28.61 | 9.30 | 1-21 | 173-251 | SCSP803280_000018581,11476,12522 | MG204152 |
| SacEXP8 | 269 |  | 29.15 | 8.96 | 1-25 | 171-253 | SCSP803280_000021378,11501,12659 | MG204161 |
| SacEXP9 | 295 |  | 30.82 | 8.82 | 1-24 | 196-279 | SCSP803280_000065540,2401,3635 | MG204123 |
| SacEXP10 | 269 |  | 29.40 | 9.43 | 1-29 | 173-254 | SCSP803280_000076988,16607,17850 | MG204166 |
| SacEXP11 | 269 |  | 29.15 | 8.87 | 1-15 | 171-253 | SCSP803280_000023980,10340,11495 | MG204178 |
| SacEXP12 | 249 |  | 25.89 | 8.36 | 1-20 | 155-232 | SCSP803280_000007238,7055,7804 | MG204128 |
| SacEXP13 | 249 |  | 26.25 | 8.31 | 1-21 | 155-232 | SCSP803280_000007238,11615,12457 | MG204127 |
| SacEXP14 | 257 |  | 27.74 | 5.99 | 1-24 | 164-245 | SCSP803280_000059448,12001,12774 | MG204194 |
| SacEXP15 | 281 |  | 29.54 | 9.49 | 1-25 | 181-264 | SCSP803280_000019559,9647,10885 | MG204157 |
| SacEXP16 | 255 |  | 26.99 | 8.41 | 1-24 | 161-238 | SCSP803280_000006655,6772,7660 | MG204122 |
| SacEXP17 | 307 |  | 32.16 | 5.45 | 1-21 | 176-257 | SCSP803280_000040437,10164,11412 | MG204135 |
| SacEXP18 | 259 |  | 28.00 | 8.33 | 1-24 | 166-243 | SCSP803280_000064058,1397,4069 | MG204115 |
| SacEXP19 | 272 |  | 28.97 | 8.01 | 1-33 | 173-255 | SCSP803280_000001972,2965,5059 | MG204176 |
| SacEXP20 | 258 |  | 27.42 | 8.93 | 1-18 | 164-241 | SCSP803280_000008764,362,1244 | MG204144 |
| SacEXP21 | 252 |  | 26.84 | 9.17 | 1-25 | 159-235 | SCSP803280_000008764,5809,6804 | MG204145 |
| SacEXP22 | 317 |  | 34.56 | 6.37 | 1-24 | 169-250 | SCSP803280_000066583,11419,12748 | MG204198 |
| SacEXP23 | 262 |  | 28.38 | 5.99 | 1-26 | 169-250 | SCSP803280_000043348,5832,6620 | MG204146 |
| SacEXP24 | 269 |  | 29.15 | 8.96 | 1-25 | 171-253 | SCSP803280_000065792,3337,4493 | MG204117 |
| SacEXP25 | 265 |  | 28.31 | 9.11 | 1-25 | 167-249 | SCSP803280_000017917,8675,12578 | MG204151 |
| SacEXP26 | 271 |  | 28.91 | 9.16 | 1-24 | 176-255 | SCSP803280_000022690,4508,5656 | MG204202 |
| SacEXP27 | 267 |  | 28.63 | 9.30 | 1-22 | 173-251 | SCSP803280_000022690,9513,10558 | MG204203 |
| SacEXP28 | 262 |  | 28.03 | 9.07 | 1-21 | 168-246 | SCSP803280_000028276,6561,7455 | MG204177 |
| SacEXP29 | 251 |  | 26.18 | 7.56 | 1-24 | 160-237 | SCSP803280_000033064,8672,9843 | MG204183 |
| SacEXP30 | 262 |  | 28.08 | 9.08 | 1-24 | 168-246 | SCSP803280_000021574,9023,9917 | MG204186 |
| SacEXP31 | 314 |  | 32.94 | 8.91 | 1-21 | 215-298 | SCSP803280_000030758,7663,8959 | MG204114 |
| SacEXP32 | 258 |  | 27.91 | 8.53 | 1-15 | 165-242 | SCSP803280_000034776,8635,11327 | MG204124 |
| SacEXP33 | 267 |  | 28.65 | 9.37 | 1-22 | 173-251 | SCSP803280_000022464,1797,2844 | MG204132 |
| SacEXP34 | 209 |  | 22.02 | 9.27 | 1-25 | 173-207 | SCSP803280_000022464,10010,10845 | MG204133 |
| SacEXP35 | 290 |  | 30.56 | 9.41 | 1-25 | 190-273 | SCSP803280_000041244,4538,5803 | MG204136 |
| SacEXP36 | 265 |  | 28.41 | 6.30 | 1-20 | 170-250 | SCSP803280_000056715,5703,6606 | MG204141 |
| SacEXP37 | 250 |  | 26.53 | 5.23 | 1-28 | 157-234 | SCSP803280_000043058,9744,10599 | MG204189 |
| SacEXP38 | 269 |  | 29.13 | 8.96 | 1-25 | 171-253 | SCSP803280_000028845,5106,6250 | MG204180 |
| SacEXP39 | 314 |  | 34.35 | 5.96 | 1-21 | 169-250 | SCSP803280_000040604,3715,5039 | MG204179 |
| SacEXP40 | 272 |  | 29.39 | 8.98 | 1-23 | 158-240 | SCSP803280_000037034,460,1880 | MG204120 |
| SacEXP41 | 278 |  | 29.61 | 5.81 | 1-26 | 183-263 | SCSP803280_000042948,3078,3981 | MG204158 |
| SacEXP42 | 262 |  | 28.09 | 9.08 | 1-25 | 168-246 | SCSP803280_000050884,6526,7420 | MG204126 |
| SacEXP43 | 249 |  | 26.77 | 8.90 | 1-23 | 155-232 | SCSP803280_000052613,7577,8479 | MG204162 |
| SacEXP44 | 290 |  | 30.54 | 9.33 | 1-18 | 190-273 | SCSP803280_000087670,673,1938 | MG204174 |
| SacEXP45 | 260 |  | 27.95 | 9.36 | 1-24 | 167-244 | SCSP803280_000050720,9191,9338 | MG204200 |
| SacEXP46 | 274 |  | 29.33 | 9.04 | 1-30 | 174-255 | SCSP803280_000098880,2995,7681 | MG204172 |
| SacEXP47 | 252 |  | 26.87 | 8.47 | 1-25 | 159-236 | SCSP803280_000041637,3813,4707 | MG204160 |
| SacEXP48 | 260 |  | 27.95 | 9.36 | 1-25 | 167-244 | SCSP803280_000064616,631,3807 | MG204201 |
| SacEXP49 | 266 |  | 28.82 | 4.88 | 1-26 | 170-251 | SCSP803280_000161764,5053,6414 | MG204121 |
| SacEXP50 | 269 |  | 29.15 | 8.87 | 1-24 | 171-253 | SCSP803280_000020687,5067,6223 | MG204182 |
| SacEXP51 | 284 |  | 30.16 | 9.75 | 1-24 | 185-267 | SCSP803280_000088941,65,1238 | MG204173 |
| SacEXP52 | 226 |  | 23.39 | 6.48 | 1-15 | 146-209 | SCSP803280_000096313,1923,2671 | MG204171 |
| SacEXP53 | 249 |  | 26.23 | 8.31 | 1-20 | 155-232 | SCSP803280_000096313,6683,7525 | MG204170 |
| SacEXP54 | 263 |  | 27.29 | 5.31 | 1-21 | 164-245 | SCSP803280_000062989,2900,4152 | MG204181 |
| SacEXP55 | 120 |  | 12.43 | 5.32 | 1-24 | 31-105 | SCSP803280_000106873,27777,3139 | MG204149 |
| SacEXP56 | 260 |  | 27.92 | 9.32 | 1-25 | 168-244 | SCSP803280_000022110,875,1763 | MG204143 |
| SacEXP57 | 314 |  | 34.13 | 6.12 | 1-24 | 169-250 | SCSP803280_000034411,4049,5369 | MG204188 |
| SacEXP58 | 250 |  | 26.50 | 8.02 | 1-21 | 156-233 | SCSP803280_000046049,1956,2840 | MG204187 |
| SacEXP59 | 252 |  | 26.83 | 9.27 | 1-24 | 159-235 | SCSP803280_000036593,5854,6810 | MG204190 |
| SacEXP60 | 316 |  | 33.49 | 9.68 | 1-33 | 218-294 | SCSP803280_000082789,4832,5932 | MG204153 |
| SacEXP61 | 259 |  | 27.46 | 8.85 | 1-18 | 165-243 | SCSP803280_000095130,1,885 | MG204112 |
| SacEXP62 | 252 |  | 26.58 | 6.01 | 1-25 | 161-238 | SCSP803280_000053114,3432,4717 | MG204196 |
| SacEXP63 | 261 |  | 27.86 | 9.42 | 1-24 | 167-245 | SCSP803280_000052621,5210,6101 | MG204130 |
| SacEXP64 | 261 |  | 28.25 | 9.46 | 1-26 | 164-245 | SCSP803280_000065298,2895,4099 | MG204154 |
| SacEXP65 | 252 |  | 26.58 | 6.01 | 1-25 | 161-238 | SCSP803280_000100614,1578,2863 | MG204167 |
| SacEXP66 | 251 |  | 26.83 | 8.87 | 1-25 | 158-235 | SCSP803280_000164264,2275,3166 | MG204148 |
| SacEXP67 | 252 |  | 26.83 | 9.27 | 1-24 | 159-235 | SCSP803280_000034663,2601,3547 | MG204175 |
| SacEXP68 | 267 |  | 28.57 | 9.22 | 1-22 | 173-251 | SCSP803280_000088702,2370,3413 | MG204159 |
| SacEXP69 | 240 |  | 25.89 | 8.64 | 1-21 | 172-223 | SCSP803280_000032256,2611,3514 | MG204169 |
| SacEXP70 | 269 |  | 29.17 | 8.96 | 1-24 | 171-253 | SCSP803280_000058057,1041,2198 | MG204142 |
| SacEXP71 | 315 |  | 32.99 | 8.78 | 1-24 | 216-299 | SCSP803280_000030877,3169,4451 | MG204156 |
| SacEXP72 | 260 |  | 27.95 | 9.36 | 1-21 | 167-244 | SCSP803280_000166501,698,3356 | MG204195 |
| SacEXP73 | 249 |  | 26.00 | 8.36 | 1-15 | 155-232 | SCSP803280_000039302,4805,5554 | MG204118 |
| SacEXP74 | 267 |  | 28.69 | 9.39 | 1-22 | 173-251 | SCSP803280_000045851,1488,2510 | MG204147 |
| SacEXP75 | 252 |  | 26.58 | 6.01 | 1-25 | 161-238 | SCSP803280_000104463,2307,3591 | MG204191 |
| SacEXP76 | 255 |  | 26.95 | 8.09 | 1-25 | 161-238 | SCSP803280_000093500,1467,2351 | MG204138 |
| SacEXP77 | 277 |  | 29.55 | 8.43 | 1-20 | 154-237 | SCSP803280_000099483,460,2222 | MG204137 |
| SacEXP78 | 272 |  | 29.34 | 9.03 | 1-28 | 158-240 | SCSP803280_000124172,1737,3183 | MG204134 |
| SacEXP79 | 254 |  | 27.05 | 8.05 | 1-25 | 160-237 | SCSP803280_000096611,2663,3542 | MG204150 |
| SacEXP80 | 259 |  | 27.00 | 6.79 | 1-21 | 162-242 | SCSP803280_000147924,2084,3399 | MG204113 |
| SacEXP81 | 281 |  | 29.60 | 8.83 | 1-23 | 188-266 | SCSP803280_000074962,903,1748 | MG204129 |
| SacEXP82 | 287 |  | 31.51 | 7.58 | 1-26 | 184-271 | SCSP803280_000047674,71,2096 | MG204125 |
| SacEXP83 | 109 |  | 11.31 | 6.18 | 1-25 | 31-105 | SCSP803280_000137179,3370,3696 | MG204164 |
| SacEXP84 | 261 |  | 27.19 | 8.85 | 1-23 | 188-259 | SCSP803280_000073906,936,1721 | MG204155 |
| SacEXP85 | 257 |  | 27.70 | 5.99 | 1-18 | 164-245 | SCSP803280_000072106,651,1424 | MG204185 |
| SacEXP86 | 314 |  | 34.29 | 5.96 | 1-24 | 169-250 | SCSP803280_000161255,1246,2570 | MG204199 |
| SacEXP87 | 271 |  | 29.53 | 8.89 | 1-30 | 174-255 | SCSP803280_000113424,273,1229 | MG204119 |
| SacEXP88 | 291 |  | 30.71 | 5.16 | 1-25 | 161-238 | SCSP803280_000145538,817,2367 | MG204131 |
| SacEXP89 | 263 |  | 27.42 | 6.79 | 1-25 | 166-246 | SCSP803280_000150752,429,1757 | MG204168 |
| SacEXP90 | 291 |  | 31.28 | 9.40 | 1-26 | 198-276 | SCSP803280_000190559,471,1346 | MG204163 |
| SacEXP91 | 120 |  | 12.38 | 4.98 | 1-24 | 31-105 | SCSP803280_000106543,330,692 | MG204184 |
| SacEXP92 | 288 |  | 31.65 | 9.43 | 1-24 | 173-254 | SCSP803280_000077471,182,1424 | MG204116 |

| **Reference strains** | **Host** | | **Expansin type** | **Size (aa)** | **NCBI number** |
| --- | --- | --- | --- | --- | --- |
| XP008373652 | *Malus domestica* | EXPLB | | 254 | XP008373652.1 |
| Q850K7 | *Oryza sativa* | EXPLB | | 256 | Q850K7.2 |
| XP002278917 | *Vitis vinifera* | EXPLB | | 255 | XP002278917 |
| Q10S70 | *Oryza sativa* | EXPLA | | 279 | Q10S70.1 |
| Q7XCL0 | *Oryza sativa* | EXPLA | | 275 | Q7XCL0 |
| Q7XT40 | *Oryza sativa* | EXPB | | 264 | Q7XT40.2 |
| Q9SHY6 | *Arabidopsis thaliana* | EXPB | | 273 | Q9SHY6.2 |
| Q0DZ85 | *Oryza sativa* | EXPB | | 272 | Q0DZ85.1 |
| AGM16366 | *Brassica rapa* | EXPB | | 265 | AGM16366.1 |
| O80622 | *Arabidopsis thaliana* | EXPA | | 253 | O80622.2 |
