# Supplementary material for: Genome-wide identification, characterization and expression profile analysis of expansins gene family in sugarcane (Saccharum spp.)
Source: PLoS One. 2018 Jan 11;13(1):e0191081. doi: 10.1371/journal.pone.0191081 (PMC5764346; doi:10.1371/journal.pone.0191081)
Supplement: S2 Table — (DOCX) [file pone.0191081.s005.docx]

| Gene | Anaerobic | Fungal elicitor | Drought | Defense and stress | Heat | Low- | SA | IAA | GA | MeJA | ethylene | ABA | Light |
| --- | --- | --- | --- | --- | --- | --- | --- | --- | --- | --- | --- | --- | --- |
|  | induction |  |  |  |  | temp |  |  |  |  |  |  |  |
| SacEXP1 | 2 | 0 | 1 | 1 | 0 | 1 | 1 | 0 | 0 | 4 | 0 | 0 | 3 |
| SacEXP2 | 1 | 0 | 1 | 0 | 1 | 0 | 0 | 1 | 0 | 2 | 0 | 3 | 15 |
| SacEXP3 | 0 | 0 | 0 | 0 | 0 | 0 | 0 | 0 | 2 | 0 | 0 | 1 | 11 |
| SacEXP4 | 0 | 0 | 0 | 1 | 1 | 0 | 1 | 0 | 0 | 4 | 0 | 0 | 5 |
| SacEXP5 | 1 | 0 | 0 | 0 | 0 | 0 | 0 | 0 | 0 | 2 | 0 | 2 | 15 |
| SacEXP6 | 0 | 0 | 0 | 0 | 1 | 1 | 0 | 1 | 2 | 0 | 0 | 0 | 9 |
| SacEXP7 | 0 | 1 | 1 | 1 | 0 | 1 | 0 | 0 | 1 | 1 | 0 | 0 | 3 |
| SacEXP8 | 1 | 0 | 0 | 0 | 0 | 0 | 1 | 3 | 1 | 0 | 0 | 0 | 14 |
| SacEXP9 | 1 | 2 | 0 | 2 | 0 | 0 | 0 | 0 | 1 | 0 | 0 | 2 | 14 |
| SacEXP10 | 0 | 0 | 1 | 0 | 0 | 0 | 1 | 0 | 0 | 0 | 0 | 0 | 13 |
| SacEXP11 | 2 | 0 | 0 | 0 | 0 | 0 | 1 | 4 | 1 | 0 | 0 | 0 | 14 |
| SacEXP12 | 1 | 2 | 0 | 0 | 0 | 0 | 0 | 0 | 1 | 2 | 0 | 2 | 12 |
| SacEXP13 | 1 | 1 | 3 | 1 | 0 | 1 | 0 | 1 | 1 | 4 | 0 | 0 | 8 |
| SacEXP14 | 2 | 1 | 2 | 1 | 0 | 0 | 0 | 2 | 0 | 4 | 0 | 1 | 6 |
| SacEXP15 | 0 | 0 | 3 | 0 | 0 | 0 | 1 | 0 | 0 | 4 | 0 | 2 | 8 |
| SacEXP16 | 2 | 0 | 2 | 0 | 0 | 0 | 2 | 0 | 3 | 2 | 0 | 0 | 4 |
| SacEXP17 | 2 | 0 | 1 | 0 | 0 | 0 | 0 | 0 | 2 | 0 | 0 | 1 | 9 |
| SacEXP18 | 0 | 0 | 2 | 2 | 0 | 0 | 1 | 0 | 4 | 4 | 1 | 3 | 9 |
| SacEXP19 | 1 | 2 | 1 | 0 | 1 | 0 | 0 | 1 | 1 | 4 | 0 | 2 | 16 |
| SacEXP20 | 0 | 0 | 0 | 0 | 0 | 0 | 0 | 0 | 0 | 2 | 0 | 0 | 4 |
| SacEXP21 | 0 | 1 | 1 | 0 | 0 | 0 | 0 | 0 | 0 | 2 |  | 1 | 11 |
| SacEXP22 | 2 | 0 | 0 | 2 | 1 | 0 | 0 | 0 | 0 | 2 | 0 | 0 | 6 |
| SacEXP23 | 0 | 2 | 0 | 0 | 0 | 0 | 2 | 2 | 0 | 2 | 0 | 0 | 11 |
| SacEXP24 | 0 | 0 | 0 | 0 | 1 | 0 | 0 | 1 | 2 | 2 | 0 | 0 | 11 |
| SacEXP25 | 1 | 0 | 0 | 0 | 0 | 0 | 0 | 0 | 0 | 2 | 0 | 2 | 3 |
| SacEXP26 | 0 | 1 | 0 | 0 | 1 | 0 | 1 | 0 | 0 | 4 | 0 | 2 | 5 |
| SacEXP27 | 0 | 1 | 1 | 1 | 1 | 1 | 0 | 0 | 1 | 2 | 0 | 0 | 2 |
| SacEXP28 | 0 | 0 | 2 | 0 | 0 | 1 | 0 | 0 | 2 | 8 | 0 | 4 | 17 |
| SacEXP29 | 0 | 0 | 2 | 1 | 1 | 1 | 2 | 0 | 1 | 0 | 0 | 4 | 15 |
| SacEXP30 | 0 | 1 | 1 | 0 | 1 | 1 | 1 | 0 | 0 | 0 | 0 | 0 | 15 |
| SacEXP31 | 1 | 0 | 3 | 0 | 1 | 3 | 2 | 1 | 1 | 4 | 0 | 0 | 11 |
| SacEXP32 | 0 | 0 | 0 | 0 | 2 | 0 | 0 | 0 | 4 | 4 | 1 | 2 | 5 |
| SacEXP33 | 0 | 1 | 1 | 2 | 1 | 1 | 0 | 0 | 1 | 2 | 0 | 0 | 3 |
| SacEXP34 | 1 | 0 | 0 | 0 | 0 | 0 | 1 | 1 | 0 | 0 | 0 | 1 | 15 |
| SacEXP35 | 0 | 1 | 1 | 0 | 1 | 0 | 1 | 1 | 0 | 2 | 0 | 0 | 8 |
| SacEXP36 | 1 | 0 | 1 | 2 | 0 | 2 | 0 | 2 | 2 | 8 | 0 | 0 | 5 |
| SacEXP37 | 0 | 1 | 0 | 0 | 0 | 0 | 1 | 0 | 0 | 4 | 0 | 0 | 4 |
| SacEXP38 | 0 | 0 | 0 | 0 | 0 | 0 | 0 | 0 | 1 | 4 | 0 | 1 | 7 |
| SacEXP39 | 2 | 0 | 0 | 2 | 0 | 0 | 0 | 0 | 0 | 2 | 0 | 0 | 7 |
| SacEXP40 | 0 | 0 | 1 | 0 | 0 | 0 | 0 | 0 | 0 | 4 | 0 | 1 | 12 |
| SacEXP41 | 1 | 1 | 2 | 0 | 2 | 2 | 1 | 1 | 0 | 6 | 0 | 2 | 7 |
| SacEXP42 | 0 | 0 | 0 | 0 | 0 | 0 | 0 | 0 | 3 | 6 | 0 | 2 | 10 |
| SacEXP43 | 0 | 0 | 0 | 0 | 0 | 0 | 1 | 0 | 1 | 0 | 0 | 0 | 12 |
| SacEXP44 | 0 | 1 | 1 | 0 | 0 | 0 | 1 | 0 | 0 | 0 | 0 | 0 | 6 |
| SacEXP45 | 0 | 0 | 1 | 0 | 0 | 0 | 0 | 2 | 0 | 8 | 0 | 0 | 12 |
| SacEXP46 | 0 | 1 | 0 | 0 | 0 | 0 | 0 | 0 | 1 | 2 | 0 | 1 | 14 |
| SacEXP47 | 0 | 0 | 1 | 0 | 0 | 1 | 0 | 0 | 2 | 6 | 0 | 2 | 11 |
| SacEXP48 | 0 | 0 | 1 | 0 | 0 | 0 | 1 | 0 | 0 | 0 | 0 | 2 | 8 |
| SacEXP49 | 2 | 0 | 1 | 2 | 0 | 0 | 1 | 0 | 1 | 2 | 0 | 0 | 7 |
| SacEXP50 | 0 | 0 | 0 | 1 | 0 | 1 | 0 | 1 | 2 | 0 | 0 | 0 | 8 |
| SacEXP51 | 0 | 0 | 1 | 0 | 0 | 0 | 0 | 0 | 0 | 0 | 0 | 0 | 0 |
| SacEXP52 | 1 | 1 | 0 | 0 | 0 | 0 | 0 | 1 | 1 | 4 | 0 | 0 | 7 |
| SacEXP53 | 2 | 0 | 3 | 1 | 0 | 1 | 0 | 0 | 0 | 4 | 0 | 0 | 10 |
| SacEXP54 | 0 | 0 | 5 | 1 | 1 | 0 | 1 | 1 | 0 | 10 | 0 | 4 | 11 |
| SacEXP55 | 0 | 0 | 0 | 0 | 0 | 0 | 0 | 0 | 0 | 0 | 0 | 0 | 0 |
| SacEXP56 | 0 | 0 | 1 | 0 | 1 | 1 | 1 | 0 | 3 | 2 | 0 | 2 | 14 |
| SacEXP57 | 0 | 0 | 0 | 0 | 2 | 0 | 1 | 1 | 1 | 4 | 0 | 0 | 10 |
| SacEXP58 | 2 | 0 | 0 | 1 | 0 | 1 | 1 | 0 | 1 | 2 | 0 | 2 | 11 |
| SacEXP59 | 0 | 1 | 0 | 0 | 1 | 0 | 1 | 0 | 0 | 2 | 0 | 1 | 10 |
| SacEXP60 | 0 | 0 | 2 | 1 | 0 | 0 | 0 | 1 | 1 | 6 | 0 | 0 | 8 |
| SacEXP61 | 0 | 0 | 0 | 0 | 0 | 0 | 0 | 0 | 0 | 0 | 0 | 0 | 0 |
| SacEXP62 | 1 | 1 | 0 | 0 | 0 | 0 | 2 | 0 | 1 | 2 | 0 | 1 | 8 |
| SacEXP63 | 0 | 1 | 1 | 0 | 1 | 1 | 1 | 0 | 0 | 0 | 0 | 0 | 13 |
| SacEXP64 | 0 | 0 | 0 | 0 | 0 | 0 | 0 | 0 | 0 | 0 | 0 | 1 | 11 |
| SacEXP65 | 1 | 1 | 0 | 0 | 0 | 1 | 2 | 0 | 1 | 2 | 0 | 1 | 7 |
| SacEXP66 | 0 | 1 | 1 | 0 | 1 | 1 | 1 | 0 | 0 | 0 | 0 | 0 | 11 |
| SacEXP67 | 1 | 0 | 4 | 1 | 0 | 0 | 0 | 0 | 0 | 2 | 0 | 3 | 12 |
| SacEXP68 | 0 | 1 | 1 | 1 | 0 | 1 | 0 | 0 | 1 | 2 | 0 | 0 | 3 |
| SacEXP69 | 3 | 0 | 3 | 0 | 1 | 0 | 0 | 0 | 1 | 2 | 0 | 2 | 5 |
| SacEXP70 | 0 | 0 | 0 | 0 | 0 | 0 | 0 | 1 | 1 | 2 | 0 | 0 | 3 |
| SacEXP71 | 0 | 0 | 2 | 1 | 0 | 0 | 0 | 1 | 1 | 0 | 0 | 2 | 15 |
| SacEXP72 | 0 | 0 | 1 | 1 | 0 | 1 | 1 | 0 | 0 | 4 | 0 | 2 | 7 |
| SacEXP73 | 1 | 1 | 0 | 0 | 0 | 0 | 0 | 1 | 1 | 4 | 0 | 1 | 10 |
| SacEXP74 | 0 | 1 | 1 | 0 | 1 | 0 | 0 | 0 | 2 | 2 | 0 | 0 | 7 |
| SacEXP75 | 1 | 1 | 0 | 0 | 0 | 0 | 2 | 0 | 0 | 1 | 0 | 1 | 8 |
| SacEXP76 | 2 | 0 | 2 | 0 | 1 | 0 | 2 | 0 | 2 | 2 | 0 | 0 | 5 |
| SacEXP77 | 0 | 0 | 0 | 0 | 0 | 0 | 1 | 0 | 1 | 2 | 0 | 2 | 4 |
| SacEXP78 | 1 | 0 | 2 | 0 | 0 | 1 | 0 | 0 | 0 | 10 | 0 | 1 | 23 |
| SacEXP79 | 2 | 0 | 1 | 1 | 0 | 1 | 1 | 0 | 0 | 4 | 0 | 0 | 3 |
| SacEXP80 | 1 | 1 | 1 | 0 | 0 | 1 | 1 | 1 | 1 | 0 | 0 | 0 | 6 |
| SacEXP81 | 1 | 0 | 1 | 0 | 2 | 0 | 0 | 0 | 0 | 2 | 1 | 0 | 4 |
| SacEXP82 | 0 | 0 | 0 | 0 | 0 | 0 | 0 | 0 | 0 | 0 | 0 | 0 | 0 |
| SacEXP83 | 0 | 1 | 1 | 0 | 2 | 0 | 1 | 0 | 1 | 2 | 1 | 0 | 9 |
| SacEXP84 | 2 | 0 | 1 | 0 | 1 | 0 | 0 | 0 | 0 | 4 | 1 | 1 | 10 |
| SacEXP85 | 0 | 0 | 2 | 0 | 0 | 0 | 0 | 1 | 0 | 4 | 0 | 1 | 6 |
| SacEXP86 | 2 | 0 | 0 | 2 | 0 | 0 | 0 | 0 | 0 | 2 | 0 | 0 | 7 |
| SacEXP87 | 0 | 0 | 1 | 0 | 0 | 0 | 0 | 0 | 0 | 0 | 0 | 0 | 3 |
| SacEXP88 | 0 | 0 | 1 | 1 | 0 | 0 | 0 | 0 | 0 | 0 | 0 | 0 | 5 |
| SacEXP89 | 1 | 0 | 1 | 0 | 1 | 0 | 0 | 0 | 0 | 0 | 0 | 0 | 4 |
| SacEXP90 | 1 | 1 | 0 | 0 | 0 | 0 | 0 | 2 | 0 | 4 | 0 | 3 | 7 |
| SacEXP91 | 1 | 1 | 0 | 0 | 0 | 0 | 1 | 0 | 0 | 0 | 0 | 0 | 3 |
| SacEXP92 | 0 | 0 | 1 | 0 | 0 | 0 | 0 | 0 | 0 | 0 | 0 | 0 | 0 |
