# Supplementary material for: Genome-wide identification, characterization and expression profile analysis of expansins gene family in sugarcane (Saccharum spp.)
Source: PLoS One. 2018 Jan 11;13(1):e0191081. doi: 10.1371/journal.pone.0191081 (PMC5764346; doi:10.1371/journal.pone.0191081)
Supplement: S3 Table — (DOCX) [file pone.0191081.s006.docx]

| **Expansin** | **Foward** | **Reverse** | **Efficiency** |
| --- | --- | --- | --- |
| BetaEXP4.2 | GCAACGAGTCCGGCAAGA | CCAGTTGGCCGGGATGA | 1.89 |
| SacEXP18 | GGGACGCTACTACTGCTACTA | CGGAGGACTAGAGAAAGAGAGA | 1.88 |
| SacEXP21 | TGCAGCTAGCCCTACAATAATC | CGCTTAGGCCTTTCCTTCTC | 1.9 |
| SacEXP22 | ATGTTGGAGCGGTAGTAGGT | ATCCATCTGGAGGATGGACTC | 1.86 |
| SacEXP39 | CTGCTGTTGGACTGGTCATTA | CCTCAATCCCATGCAAATGATAC | 1.87 |
| SacEXP49 | GGAACCCTCTTCGTTGTTGTA | GATCAGAGAAGGAAAGGCCTAAG | 1.82 |
| SacEXP57 | CAAACAAACGGGACAGAGTTTAC | TCTCTGGTTAGGAGGACGATAC | 1.94 |
| SacEXP59 | CACCGTGATAACTGCCAAGAG | CATGGCTACGGAGCAAATCA | 1.91 |
| SacEXP78 | GTCAGCTCAGGTATTCGATCAG | CCTCTTATCCATCTCCACCTTG | 1.92 |
| SacEXP86 | GACCGTGGTCGGTTAATTAGTAT | CTGGTGGCCAAGGACATTAT | 1.9 |
